# Draft Genome Sequence of *Providencia heimbachae*, Isolated from a Diabetic Foot Ulcer

**DOI:** 10.1128/genomeA.00276-16

**Published:** 2016-04-14

**Authors:** Joanne Jneid, Samia Benamar, Isabelle Pagnier, Pierre-Yves Levy, Jean-Philippe Lavigne, Bernard La Scola

**Affiliations:** a Faculté de Médecine, Unité de Recherche sur les Maladies Infectieuses et Tropicales Emergentes, CNRS, UMR 6236–IRD 198, Aix-Marseille Université, Marseille, France; b Fédération de Bactériologie-Virologie-Hygiène, Assistance Publique Hôpitaux de Marseille, Hôpital de la Timone, Marseille, France; c Service de Microbiologie, CHU Caremeau, Nîmes, France; d INSERM, U1047, Université de Montpellier, Nîmes, France

## Abstract

*Providencia* spp. are ubiquitous Gram-negative bacteria of the family *Enterobacteriaceae* that are common opportunistic pathogens. In the present work, we have sequenced, annotated, and compared the draft genome of *Providencia heimbachae*, which was recovered from a diabetic foot ulcer. It is composed of 4.22 Mb and encodes 3,843 protein-coding genes and 79 RNA genes, including 11 rRNA genes.

## GENOME ANNOUNCEMENT

*Providencia* spp. are ubiquitous Gram-negative bacteria in the *Enterobacteriaceae* family that are often opportunistic pathogens. Members of this genus have been identified as part of the normal human gut flora (1) and have been associated with numerous animals worldwide (2, 3). In 1986, Muller et al. (4) characterized a new group of *Providencia* and described *Providencia heimbachae* on the basis of biochemical and DNA hybridization tests*.* The first isolate was obtained from the stool of a 23-year-old woman in Seattle–King County, Washington, USA, who suffered from unexplained diarrhea (5).

In the present work, we have sequenced, annotated, and compared the draft genome of *P. heimbachae*, isolated from a diabetic foot ulcer in 2014 and deposited in our collection CSUR under number P2169. The genomic DNA was sequenced using a combination of paired-end libraries (average insert size of 250 bp) and mate-pair (2.5-kb) libraries on the Illumina MiSeq. The generated reads were assembled using the SPADES genome assembler (6). The assembled genome counts eight contigs regrouped in seven scaffolds. The estimated genome size was 4,220,813 bp with a G+C content of 40.31%, which is identical to *Providencia rettgeri* strain H1736 (GenBank: NZ_CVLT01000017). Both the estimated genome size and the G+C content are in the range of the species of the *Providencia* genus.

A total of 3,843 protein-coding sequences were predicted, forming the sequence coding and representing 85.71% of the genome size. We identified 79 structural RNAs (8 genes are 5S rRNA, 1 gene is 16S rRNA, 2 genes are 23S rRNA, and 68 genes are tRNA genes) using tRNAscan-SE version 1.3 (7) and RNAmmer (8). A homology search against the public protein database from NCBI using the BLASTp program identified 455 hypothetical proteins; 1,094 proteins associated to virulence were found, representing 28.46% of the total genes.

### Nucleotide sequence accession numbers.

This whole-genome project has been deposited at DDBJ/EMBL under the accession numbers FIZD01000001 to FIZD01000008.

## References

[1] Peterson J, Garges S, Giovanni M, McInnes P, Wang L, Schloss JA, Bonazzi V, McEwen JE, Wetterstrand KA, Deal C, Baker CC, Di Francesco V, Howcroft TK, Karp RW, Lunsford RD, Wellington CR, Belachew T, Wright M, Giblin C, David H, Mills M, Salomon R, Mullins C, Akolkar B, Begg L, Davis C, Grandison L, Humble M, Khalsa J, Little AR, Peavy H, Pontzer C, Portnoy M, Sayre MH, Starke-Reed P, Zakhari S, Read J, Watson B, Guyer M, NIH HMP Working Group 2009 The NIH human microbiome project. Genome Res 19:2317–2323. 19819907 10.1101/gr.096651.109 PMC2792171

[2] Muller HE 1983 *Providencia friedericiana*, a new species isolated from penguins. Int J Syst Bacteriol 33:709–715. doi:10.1099/00207713-33-4-709.

[3] Foti M, Giacopello C, Bottari T, Fisichella V, Rinaldo D, Mammina C 2009 Antibiotic resistance of Gram negatives isolates from loggerhead sea turtles (*Carettacaretta*) in the Central Mediterranean Sea. Mar Pollut Bull 58:1363–1366. doi:10.1016/j.marpolbul.2009.04.020. 19473669

[4] Muller HE, O’Hara MC, Fanning GR, Brenner FW, Swenson JM, Brenner DJ 1986 *Providencia heimbachae*, a new species of *Enterobacteriaceae* isolated from animals. Int J Syst Bacteriol 36:252–256. doi:10.1099/00207713-36-2-252.

[5] Mohr O’Hara C, Steigerwalt AG, Green D, McDowell M, Hill BC, Brenner DJ, Miller JM 1999 Isolation of *Providencia heimbachae* from human feces. J Clin Microbiol 37:3048–3050. 10449504 10.1128/jcm.37.9.3048-3050.1999 PMC85453

[6] Bankevich A, Nurk S, Antipov D, Gurevich AA, Dvorkin M, Kulikov AS, Lesin VM, Nikolenko SI, Pham S, Prjibelski AD, Pyshkin AV, Sirotkin AV, Vyahhi N, Tesler G, Alekseyev MA, Pevzner PA 2012 SPAdes: a new genome assembly algorithm and its applications to single-cell sequencing. J Comput Biol 19:455–477. doi:10.1089/cmb.2012.0021. 22506599 PMC3342519

[7] Lowe TM, Eddy SR 1997 tRNAscan-SE: a program for improved detection of transfer RNA genes in genomic sequence. Nucleic Acids Res 25:955–964. doi:10.1093/nar/25.5.0955. 9023104 PMC146525

[8] Lagesen K, Hallin P, Rødland EA, Staerfeldt HH, Rognes T, Ussery DW 2007 RNAmmer: consistent and rapid annotation of ribosomal RNA genes. Nucleic Acids Res 35:3100–3108. doi:10.1093/nar/gkm160. 17452365 PMC1888812

